# COMMENTARY ON: EXPLORING EXERCISE INTOLERANCE IN ADULT PATIENTS WITH PERSISTENT POST-CONCUSSION SYMPTOMS AFTER MILD TRAUMATIC BRAIN INJURY

**DOI:** 10.2340/jrm.v58.45050

**Published:** 2026-02-26

**Authors:** Jun TAO, Hongjian LI, Wenqi FENG

**Affiliations:** Department of Rehabilitation Medicine, Yibin Hospital of Traditional Chinese Medicine, Sichuan, 644000, China

*To the Editor,*

Valaas and colleagues (1) take on a timely question in rehabilitation: how frequently adults with persistent post-concussion symptoms (PCS) after mild traumatic brain injury (mTBI) show exercise intolerance (EI), and which factors track with it. We welcome this focus. Three analytic choices, however, deserve a closer look because they cloud interpretation and can be remedied without altering the study’s aims.

First, the abstract reports the association between higher pre-test heart rate and a higher Buffalo Concussion Treadmill Test (BCTT) symptom threshold with a coefficient of 0.34 and a 95% confidence interval of 0.17 to −0.50 (1). The interval is internally inconsistent; the bounds are reversed and the sign is incompatible with the stated direction. In the main text and Table III, the same effect is reported as 0.17 to 0.50 with *p* < 0.001, which is coherent and indicates a positive association (1). A concise erratum that corrects the abstract and checks for internal agreement across sections would prevent confusion and preserve confidence in the statistical reporting.

Second, time since injury differs between groups yet is omitted from the logistic model relating the Rivermead Post Concussion Symptoms Questionnaire (RPQ) score to EI. Table I indicates a meaningful gap in months since injury (6.95 vs 9.44; *p* = 0.010) between EI and exercise-tolerant participants (1). Because recovery after traumatic brain injury is time dependent, not adjusting for this covariate risks attributing the effect of injury chronicity to symptom burden. The reported odds ratio of 1.07 per RPQ point may therefore be inflated by recovery stage. Including months since injury as a covariate, and verifying the result in stratified or sensitivity analyses, would clarify whether RPQ independently predicts EI.

Third, the linear regression of the BCTT symptom threshold uses only the 81% who were EI and excludes the 19% who completed the test without symptom exacerbation (1). For these exercise-tolerant participants, the threshold lies at or beyond the stopping criterion and is unobserved, which makes the outcome right censored. Discarding them can bias coefficients for predictors such as pre-test heart rate and anxiety and narrows applicability to the full clinic population. A straightforward remedy is to treat the threshold as a censored outcome and analyse it with Tobit or survival methods, coding those who did not reach a symptom limit as right censored at the test maximum or at 90% of age-predicted heart rate. At minimum, the manuscript should state clearly that inferences from the threshold model apply only to the EI subgroup.

Taken together, these are tractable problems: a corrected confidence interval, a model that respects time since injury, and an analysis that honours censoring would materially improve interpretability. None of these changes alters the clinical message that graded, subthreshold aerobic exercise has growing support in the management of persistent PCS (2–4). Evidence from randomized and systematic work shows that carefully prescribed exercise below the symptom threshold can accelerate recovery and reduce the risk of prolonged symptoms (2, 3). The observation that a large majority of patients in this cohort met criteria for EI underscores the value of rigorous modelling, as these results are used to guide return-to-exercise decisions. Addressing the points mentioned would help the field judge the relation between symptom burden and exercise capacity with greater precision and, ultimately, translate testing into safer, more effective rehabilitation.
