# Supplementary material for: COMMENTARY ON: EXPLORING EXERCISE INTOLERANCE IN ADULT PATIENTS WITH PERSISTENT POST-CONCUSSION SYMPTOMS AFTER MILD TRAUMATIC BRAIN INJURY
Source: J Rehabil Med. 2026 Feb 26;58:45050. doi: 10.2340/jrm.v58.45050 (PMC12949431; doi:10.2340/jrm.v58.45050)
Supplement: Related texts

# REPLY TO THE COMMENTARY ON: EXPLORING EXERCISE INTOLERANCE IN ADULT PATIENTS WITH PERSISTENT POST-CONCUSSION SYMPTOMS AFTER MILD TRAUMATIC BRAIN INJURY

**Authors:** Lars-Johan V. VALAAS

**Affiliations:** Department of Rehabilitation Medicine and Rehabilitation, Oslo University Hospital, Oslo, Norway. Faculty of Medicine, Institute of Health and Society, University of Oslo, Oslo, Norway

Thank you for your interest in our paper and for your reflections. This paper as stated in the title aims to explore exercise intolerance, which we believe is a little understood concept in the clinic, especially for patients with persistent symptoms, and research is warranted.

Thank you for also noticing the incidental extra hyphen.

As reported, we used a logistic model adjusted for sex and age to examine the association between Buffalo Concussion Treadmill Test (BCTT) outcome (exercise intolerant vs exercise tolerant) and symptom burden. This analysis was designed to describe associations rather than establish causality, and future studies are needed to elucidate causal and mediating pathways, including the potential role of time since injury, underlying physiology, appropriate psychological measurements, and exercise capacity - which is very difficult to measure in this patient group.

Furthermore, in a cross-sectional study, time since injury may also reflect selection mechanisms, as subgroups of patients may be referred to hospital at different time points (e.g., directly from emergency department due to injury characteristics or later from general practitioner due to persistent symptoms). Because analyses and interpretation of these factors were beyond the scope of the study, time since injury was not included in this model. That said, including time since injury as a covariate did not substantial change the model (OR: RPQ = 1.056 [p = 0.046], sex [male] = 1.22 [p = 0.722], age = 0.94 [p=0.016], months since injury = 0.88 [p = 0.056], nor the conclusions. However, months since injury in this context should be interpreted with caution, and future studies should examine the temporal development of exercise intolerance in patients with persistent symptoms.

Regarding the linear regression of the BCTT symptom threshold, this analysis was intentionally restricted to participants who met the exercise intolerance criteria. We used the BCTT both to classify patients as exercise tolerant or exercise intolerant, and in the latter group, to assess symptom threshold. As clearly stated in the Methods and Results, only exercise intolerant patients were included, and symptom threshold was observed for all patients in that group. Approaches for right-censored outcomes (e.g., Tobit or time-to-event models) are primarily relevant for a different estimand with the full cohort, where exercise tolerant patients would contribute with censored thresholds. Including exercise tolerant patients in this analysis would therefore change the research question and estimand from identifying associated variables of symptom threshold within exercise intolerance. As stated, we explored exercise intolerance, not exercise capacity.

For these reasons, we do not consider the latter two points to constitute methodological issues within the scope of the stated aims. At the same time, based on our analysis in which level of physical activity level was not a significant variable, we agree that further studies, conducted in other centers and countries would be valuable.
